# 

**Published:** 1885-10

**Authors:** 


### CONTENTS.

### PROCEEDINGS.

Association of Dental College Faculties........ 481
American Dental Association—Twenty-fifth Annual Meeting........ 488

SALMAGUNDI........ 526

### EDITORIAL.

Ohio State Dental Society........ 529
American Agriculturist, for the Farm, Garden, and Household ...... 532

